# Association between treatment setting and outcomes among oregon medicaid patients with opioid use disorder: a retrospective cohort study

**DOI:** 10.1186/s13722-022-00318-1

**Published:** 2022-08-19

**Authors:** Daniel M. Hartung, Sheila Markwardt, Kirbee Johnston, Jonah Geddes, Robin Baker, Gillian Leichtling, Christi Hildebran, Brian Chan, Ryan R. Cook, Dennis McCarty, Udi Ghitza, P. Todd Korthuis

**Affiliations:** 1 Oregon State University College of Pharmacy, Portland, OR USA; 2 Oregon Health & Science University, Biostatistics and Design Program, Portland, OR USA; 3 PSU-OHSU School of Public Health, Portland, OR USA; 4 Comagine Health, Portland, OR USA; 5 Oregon Health & Science University, Section of Addiction Medicine, Portland, OR USA; 6 National Institute On Drug Abuse (NIDA), Center for the Clinical Trials Network (CCTN), Bethesda, ML USA; 7 College of Pharmacy, Oregon State University @ Oregon Health & Science University, Robertson Collaborative Life Sciences Building (RLSB), 2730 S Moody Ave., CL5CP, Portland, OR 97201-5042 USA

**Keywords:** Treatment, Opioid use disorder, Medications for opioid use disorder, Residential treatment

## Abstract

**Background:**

Residential treatment is a common approach for treating opioid use disorder (OUD), however, few studies have directly compared it to outpatient treatment. The objective of this study was to compare OUD outcomes among individuals receiving residential and outpatient treatment.

**Methods:**

A retrospective cohort study used linked data from a state Medicaid program, vital statistics, and the Substance Abuse and Mental Health Services Administration (SAMHSA) Treatment Episodes Dataset (TEDS) to compare OUD-related health outcomes among individuals treated in a residential or outpatient setting between 2014 and 2017. Multivariable Cox proportional hazards and logistic regression models examined the association between treatment setting and outcomes (i.e., opioid overdose, non-overdose opioid-related and all-cause emergency department (ED) visits, hospital admissions, and treatment retention) controlling for patient characteristics, co-morbidities, and use of medications for opioid use disorders (MOUD). Interaction models evaluated how MOUD use modified associations between treatment setting and outcomes.

**Results:**

Of 3293 individuals treated for OUD, 957 (29%) received treatment in a residential facility. MOUD use was higher among those treated as an outpatient (43%) compared to residential (19%). The risk of opioid overdose (aHR 1.39; 95% CI 0.73–2.64) or an opioid-related emergency department encounter or admission (aHR 1.02; 95% CI 0.80–1.29) did not differ between treatment settings. Independent of setting, MOUD use was associated with a significant reduction in overdose risk (aHR 0.45; 95% CI 0.23–0.89). Residential care was associated with greater odds of retention at 6-months (aOR 1.71; 95% CI 1.32–2.21) but not 1-year. Residential treatment was only associated with improved retention for individuals not receiving MOUD (6-month aOR 2.05; 95% CI 1.56–2.71) with no benefit observed in those who received MOUD (aOR 0.75; 95% CI 0.46–1.29; interaction p = 0.001).

**Conclusions:**

Relative to outpatient treatment, residential treatment was not associated with reductions in opioid overdose or opioid-related ED encounters/hospitalizations. Regardless of setting, MOUD use was associated with a significant reduction in opioid overdose risk.

**Supplementary Information:**

The online version contains supplementary material available at 10.1186/s13722-022-00318-1.

## Background

Approximately 2.7 million Americans have an opioid use disorder (OUD), yet fewer than 20% report receiving treatment [1, 2]. Initial treatment setting for OUD varies, but may involve medically supervised withdrawal (i.e. detoxification) in an inpatient or residential facility; treatment may also be initiated as an outpatient. The American Society of Addiction Medicine (ASAM) Criteria are widely used guidelines for level of care placement for patients with substance use disorders [3]. Studies suggest that patients who receive an insufficient intensity of treatment have worse outcomes compared to those who are triaged appropriately [4, 5]. While it is firmly established that medications for opioid use disorder (MOUD) such as buprenorphine and methadone are very effective at reducing opioid-related harms (e.g. overdose, death, infectious disease) and improving other addiction outcomes [6, 7], the optimal setting in which to initiate treatment remains unclear [8, 9].

Historically, spending on residential care has been a major cost center for the treatment of substance use disorders, accounting for 26–37% of spending between 2006 and 2015 [10]. While the role of outpatient care has increased, one in four adults continue to receive addiction treatment in a residential treatment facility [11]. Although residential treatment may have some advantages over outpatient care for individuals with OUD such as providing structured environment free from substances and the provision of clinical services like withdrawal support, evidence that it improves outcomes is sparse [12, 13]. Most notably, individuals entering residential or inpatient treatment are less likely to be offered ongoing outpatient care, including MOUD, which is associated with reduced rates of return to use [14, 15]. Models of care that link inpatient or residential withdrawal management induction to outpatient treatment improve outcomes.[16, 17]. Yet, fewer than half of patients who receive medically supervised withdrawal are transitioned to outpatient MOUD treatment, leading to increased risk of overdose [18–20].

Three recent studies examined the comparative effectiveness of treatment setting on OUD-related outcomes [20–22]. In a cohort of more than 30,000 patients undergoing medically managed inpatient detoxification in Massachusetts, Walley et al. found that subsequent MOUD was associated with a large reduction in opioid-related mortality (adjusted hazard ratio = 0.31), especially when combined with continued use within a residential treatment setting (adjusted hazard ratio = 0.14) compared to no further treatment [20] Yet, studies suggest fewer than a third of residential treatment settings offer opioid agonist therapy (i.e., buprenorphine or methadone) [23, 24].

Growing evidence suggests that outpatient treatment, when coupled with MOUD, may be superior to other treatment settings. Studies among commercial or Medicare Advantage enrolled patients demonstrate that outpatient treatment involving MOUD is associated with fewer overdoses, readmissions, or subsequent inpatient detoxification stays compared to inpatient detoxification or residential treatment [21, 22]. However, these recent studies comparing treatment settings have not focused on individuals with Medicaid, which has a critical role in responding to our nation’s opioid crisis. Collectively, state Medicaid programs cover 38% of individuals with OUD and finance more than half of all treatment in the US [25]. Additionally, those enrolled in Medicaid also have a considerably higher risk of opioid overdose [26]. Using a dataset linking administrative Medicaid claims, Substance Abuse and Mental Health Services Administration (SAMHSA) Treatment Episodes Dataset (TEDS), and vital statistics data from the state of Oregon, this analysis compared clinical outcomes for Medicaid beneficiaries with OUD receiving residential or outpatient treatment.

## Material and methods

### Data sources and study sample

The National Drug Abuse Treatment Clinical Trials Network (UG1DA015815) In and Out study (CTN-0086) was a retrospective cohort study comparing outcomes among individuals with OUD treated in different settings based on a secondary analysis of linked Medicaid, TEDS, and vital statistics data from Oregon. TEDS and Medicaid records were linked on the recipients’ Medicaid ID. TEDS data are collected by states and maintained by SAMHSA to track admissions (TEDS-A) to publicly funded substance use treatment facilities. For each admission, TEDS includes individual-level information describing substances used, routes of administration, frequency of use, and age of first use as well as demographics, and treatment-related characteristics (e.g. level of care). Oregon’s Medicaid data were also linked to the state’s death certificate data to identify opioid-related overdose fatalities. This linked dataset has been described and used in other studies related to opioid overdose [27–30].

### Cohort and independent variable definitions

Individuals were included if they had procedure codes indicating a residential treatment stay or an outpatient treatment encounter (Additional file 1: Table S1) and diagnosis of OUD (Additional file 1: Table S2) between July 2014 to June 2017 [31, 32]. For those with multiple episodes, we selected the first chronologically and designated that as their index treatment encounter. We excluded individuals with fewer than 180 days of Medicaid enrollment prior to their treatment episode to ensure a sufficient period to capture baseline clinical characteristics. We used the 180-days preceding their index admission to assess baseline clinical characteristics using diagnoses described in the Elixhauser comorbidity index [33–35]. The Elixhauser comorbidity index contains a standardized set of thirty categories of behavioral (e.g. depression, ‘drug abuse,’ psychoses) and physical health (e.g. congestive heart failure, diabetes, liver disease) conditions based on International Classification of Disease (ICD) diagnosis codes and has been shown to predict in-hospital mortality and readmissions (Additional file 1: Table S3) [36].

We used linked TEDS data to ascertain additional details about their OUD such as opioid type (e.g. heroin, prescription opioids), other substances used (e.g. stimulants, alcohol), and other features of their OUD such as years of misuse, opioid injection, and frequency (e.g. daily, 3–6 times/week, 1–2 times/week, 1–3 times/month, no use in past month), whether they injected opioids, and frequency of use. We included patients if they had a TEDS admission record at any point during the baseline period and up to 7 days following their Medicaid index date. TEDS admission and Medicaid treatment episode index dates were identical for 60% episodes (72% of outpatient episodes and 42% of residential episodes). For individuals with multiple TEDS admissions, we used all admissions to quantify addiction characteristics with respect to substances used, frequency, and route. We excluded individuals whose TEDS admission record did not indicate opioid (heroin, prescription opioid, non-prescription methadone) as a problem substance (5.4% of individuals with linked TEDS data and an outpatient or residential episode).

We defined treatment-related (i.e. baseline) use of MOUD with Medicaid claims occurring during and up to 30 days after their residential index episode. Because an index outpatient treatment episode could persist for a longer period of time, we defined treatment-related MOUD for only those episodes where MOUD was present within the first 30 days of their index outpatient treatment date. MOUD included methadone dispensed through an opioid treatment program, office-based buprenorphine, and extended-release injectable naltrexone (XR-NTX). Buprenorphine was identified using National Drug Codes in outpatient pharmacy prescription claims (Additional file 1: Table S4). MOUD treatment with methadone or XR-NTX was identified using CPT codes in medical claims data (Additional file 1: Table S4).

### Outcome definitions

The primary outcome was fatal or non-fatal opioid overdose. Fatal opioid overdoses were identified using Oregon’s vital statistics death data using ICD-10 codes (Additional file 1: Table S5). Potential non-fatal opioid overdoses were identified using Medicaid claims as an emergency department encounter or hospitalization (ED/hospitalization) with ICD diagnostic codes for an opioid poisoning (Additional file 1: Table S5). These codes have been validated in other Medicaid datasets and endorsed by the Centers for Disease Control and Prevention [37, 38].

We also examined three other secondary outcomes: non-overdose opioid-related ED/hospitalizations (e.g. opioid dependence; Additional file 1: Table S5), all cause ED/hospitalizations, and overall treatment retention. We defined retention as number of days from index episode until the day when all treatment across any treatment setting was discontinued. We defined discontinuation as having a 60-day gap without any claims for substance use disorder treatment services including provision of MOUD [32, 39].

### Statistical analysis

We used multivariate Cox proportional hazards regression models to estimate the associations between treatment setting and overdose and ED/hospitalization outcomes. Following their index treatment episode, individuals were followed until they were censored by loss of Medicaid enrollment (one-month gap in enrollment) or the end of the study (December 2017). We included all baseline covariates for statistical adjustment. We chose the Elixhauser comorbidity composite score over the individual condition variables to increase the events-per-variable ratio and stabilize model estimates. Included covariates were: all baseline demographics (age, race, sex), Elixhauser comorbidity score, TEDS OUD characteristics (substances used, injection drug use, frequency of use), MOUD use, and medically managed withdrawal immediately preceding their treatment episode (Additional file 1: Table S1). We also adjusted for all-cause and opioid-related ED/hospitalizations occurring in the 180 prior to their index treatment episode.

The follow-up period was from index treatment episode date until an event occurred or the individual was censored by loss of Medicaid enrollment or the end of the study period. The variance inflation factor assessed multicollinearity. Cox proportional hazard models met all necessary assumption except all cause ED/hospitalization model which violated the proportional hazards assumption. To resolve this, we split this analysis into two separate follow-up periods (time 0 to day 13, day 14 until end of study).

For the analysis of treatment retention, we used a subset of individuals with at least one year of Medicaid enrollment after their index treatment date to evaluate the association between treatment setting and retention. Using the same adjustment variables included in the Cox proportional hazard models, we used multivariable logistic regression to evaluate the association between treatment setting and retention at six and twelve months.

Our main hypothesis concerned identifying clinical benefits of residential over outpatient treatment, independent of potential unbalanced confounders (e.g. MOUD use). However, because MOUD is a vital component of OUD treatment, we also examined how its use modified potential associations between treatment setting and all opioid-related outcomes. To do this, we included the interaction between MOUD and treatment setting in our models and report treatment setting associations stratified by MOUD. All analyses used SAS (version 9.4) and considered two-sided p-values < 0.05 to be statistically significant. This study was deemed non-human subjects research by the Oregon Health & Science University Institutional Review Board.

## Results

### Study sample characteristics

**Fig. 1:**
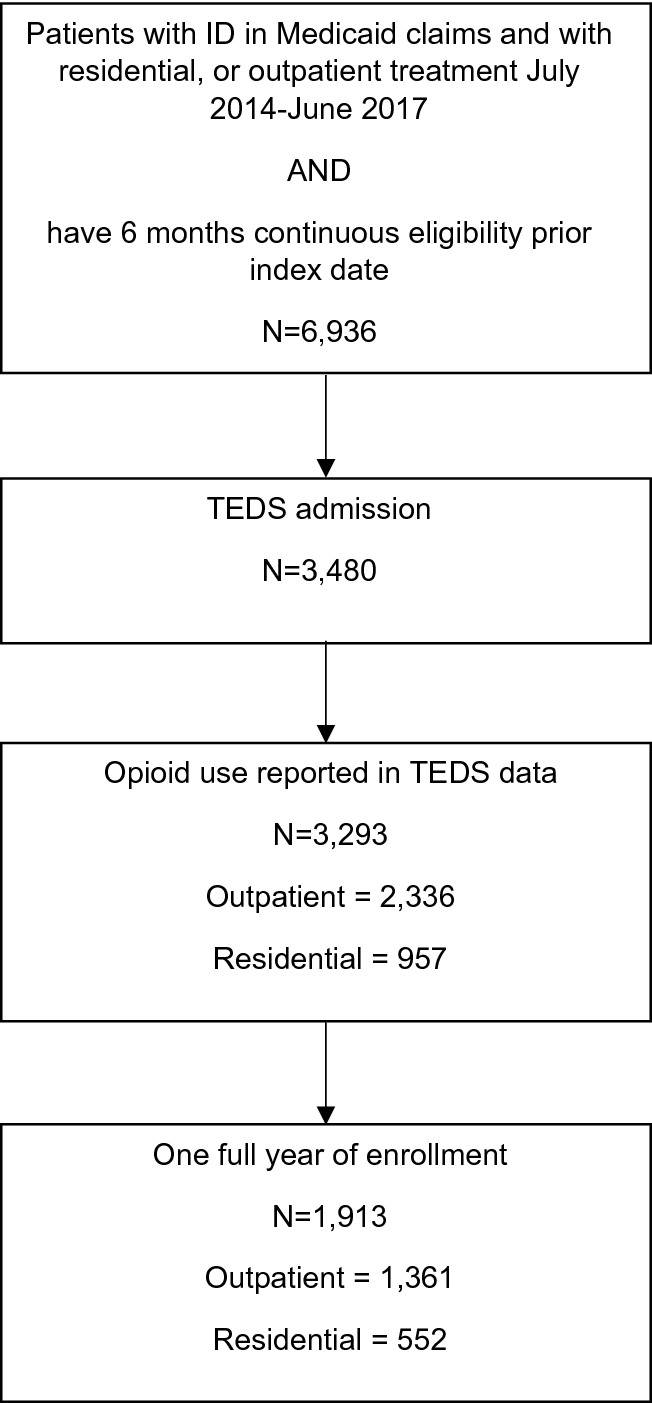
Flow diagram of patients selected for study inclusion

We identified 3293 individuals receiving treatment for OUD that met inclusion criteria (Fig. 1). Table 1 summarizes demographic, co-morbidities, treatment, and addiction-related characteristics. There were 957 individuals with an index episode in a residential facility, and 2336 treated as outpatients. Most individuals were White (74%), male (54%), and between the ages of 18 and 39 (78%). Aside from drug misuse, which was present in all patients, the most common Elixhauser conditions were alcohol misuse (20%), depression (17%), and psychoses (10%). In general, individuals treated in a residential facility had higher rates of comorbidity. Heroin was the most commonly used opioid (71%). About 39% of individuals reported a problem with prescription opioids, 39% with stimulants, and 20% with alcohol. Nearly half of individuals reported injection drug use and 57% reported daily opioid use.

**Table 1:** Patient and treatment characteristics, N = 3293

	Total (n = 3293)	Outpatient (n = 2336)	Residential (n = 957)	p-value
Female	1511 (45.9%)	1040 (44.5%)	471 (49.2%)	0.01
Race				
White	2445 (74.2%)	1740 (74.5%)	705 (73.7%)	0.10
Black or African American	46 (1.4%)	38 (1.6%)	8 (0.8%)	
Alaska Native/American Indian	105 (3.2%)	76 (3.3%)	29 (3.0%)	
Other single race	118 (3.6%)	90 (3.9%)	28 (2.9%)	
Two or more races	579 (17.6%)	392 (16.8%)	187 (19.5%)	
Ethnicity				
Hispanic or Latinx	247 (7.5%)	161 (6.9%)	86 (9.0%)	< 0.01
Not Hispanic or Latino	2915 (88.5%)	2063 (88.3%)	852 (89.0%)	
Unknown/Not Reported	131 (4.0%)	112 (4.8%)	19 (2.0%)	
Age				
18–29	1520 (46.2%)	1003 (42.9%)	517 (54.0%)	< 0.01
30–39	1039 (31.6%)	734 (31.4%)	305 (31.9%)	
40 +	734 (22.3%)	599 (25.6%)	135 (14.1%)	
Elixhauser conditions (prevalence > 3% presented)				
Alcohol misuse	667 (20.3%)	404 (17.3%)	263 (27.5%)	< 0.01
Anemia deficiency	99 (3.0%)	70 (3.0%)	29 (3.0%)	0.96
Chronic pulmonary disease	302 (9.2%)	207 (8.9%)	95 (9.9%)	0.34
Depression	545 (16.6%)	314 (13.4%)	231 (24.1%)	< 0.01
Drug misuse	3292 (100.0%)	2335 (100.0%)	957 (100.0%)	-
Hypertension	317 (9.6%)	242 (10.4%)	75 (7.8%)	0.03
Liver disease	175 (5.3%)	119 (5.1%)	56 (5.9%)	0.38
Fluid and electrolyte disorders	179 (5.4%)	115 (4.9%)	64 (6.7%)	0.04
Other neurological disorders	231 (7.0%)	139 (6.0%)	92 (9.6%)	< 0.01
Psychoses	332 (10.1%)	170 (7.3%)	162 (16.9%)	< 0.01
Elixhauser score, mean (SD)	-7.0 (5.0)	-6.8 (4.8)	-7.4 (5.4)	< 0.01
MOUD use				
Any	1177 (35.7%)	999 (42.8%)	178 (18.6%)	< 0.01
Methadone	362 (11.0%)	270 (11.6%)	92 (9.6%)	0.11
Buprenorphine	827 (25.1%)	739 (31.6%)	88 (9.2%)	< 0.01
XR naltrexone	6 (0.2%)	2 (0.1%)	4 (0.4%)	0.04
Substance used				
Heroin	2322 (70.5%)	1566 (67.0%)	756 (79.0%)	< 0.01
Prescription opioids	1272 (38.6%)	980 (42.0%)	292 (30.5%)	< 0.01
Non-prescription methadone	80 (2.4%)	67 (2.9%)	13 (1.4%)	0.01
Alcohol	659 (20.0%)	482 (20.6%)	177 (18.5%)	0.16
Stimulant	1274 (38.7%)	760 (32.5%)	514 (53.7%)	< 0.01
Injection opioid use	1637 (49.7%)	1058 (45.3%)	579 (60.5%)	< 0.01
Frequency of use				
None in past month	711 (21.6%)	525 (22.5%)	186 (19.4%)	< 0.01
Use in past week/month	694 (21.1%)	452 (19.4%)	242 (25.3%)	
Daily	1888 (57.3%)	1359 (58.2%)	529 (55.3%)	
Prior inpatient medically supervised withdrawal	570 (17.3%)	154 (6.6%)	416 (43.5%)	< 0.01
Prior all-cause ED/hospitalization	1747 (53.1%)	1143 (48.9%)	604 (63.1%)	< 0.01
Prior opioid-related ED/hospitalization	279 (8.5%)	149 (6.4%)	130 (13.6%)	< 0.01

*CI* confidence interval, *ED* emergency department encounter, *MOUD* medications for opioid use disorder, *SD* standard deviation

Overall, 17% of patients had inpatient medically managed withdrawal immediately preceding their treatment episode, however this was more than sixfold more likely in those receiving residential treatment (44% vs 7%). About one-third (36%) of individuals received MOUD treatment during their index treatment episode. Receipt of MOUD was higher among those treated as outpatients (43% vs. 19%). The most prevalent type of MOUD was buprenorphine (25%).

### Opioid-related and healthcare utilization outcomes

Table 2 summarizes Cox proportional hazard models comparing outcomes across treatment settings. Of the 56 individuals experiencing an opioid overdose, 24 (2.5%) were among those receiving residential treatment and 32 (1.4%) received outpatient treatment; 25 of these events were fatal. The risk of opioid overdose did not differ significantly between individuals who were treated in a residential setting (adjusted hazard ratio [aHR] 1.39; 95% confident interval [CI] 0.73 to 2.64) compared to those treated as outpatients. Receipt of MOUD was associated with a reduction in opioid overdose (aHR 0.45; 95% CI 0.23 to 0.89). Table 3 summarizes the interaction models and adjusted ORs stratified by MOUD use. The interaction between MOUD and setting did not meet the threshold for statistical significance.

**Table 2:** Cox proportional hazards regression models of overdose and emergency department (ED) / hospitalization outcomes (N = 3293)

	Opioid OD	*Opioid-related* *ED visit/hospitalization*	All cause ED visit/hospitalization
	Number with event (%)		
Treatment setting			
Outpatient	32 (1.4%)	297 (12.7%)	1446 (61.9%)
Residential	24 (2.5%)	147 (15.4%)	717 (74.9%)
	Adjusted HR (95% CI)		
Treatment setting			
Outpatient	ref	ref	–
Residential	1.39 (0.73–2.64)	1.02 (0.80–1.29)	–
Outpatient < 14 days post-admission	–	–	Ref
Residential < 14 days post-admission	–	–	2.48 (1.98–3.11)
Outpatient 14 + days post-admission	–	–	Ref
Residential 14 + days post-admission	–	–	1.25 (1.12–1.41)
Gender			
Male	Ref	Ref	Ref
Female	0.84 (0.48–1.49)	1.44 (1.19–1.75)	1.36 (1.24–1.48)
Race			
White	Ref	Ref	Ref
Black or African American	0.83 (0.10–6.89)	0.53 (0.20–1.42)	1.29 (0.92–1.81)
Alaska Native/American Indian	1.62 (0.39–6.83)	1.38 (0.86–2.20)	1.13 (0.89–1.43)
Other single race	1.92 (0.58–6.30)	1.59 (1.01–2.51)	1.08 (0.85–1.36)
Two or more races	1.16 (0.60–2.25)	0.91 (0.72–1.16)	1.04 (0.93–1.17)
Age			
18–29	Ref	Ref	Ref
30–39	1.57 (0.80–3.07)	0.92 (0.73–1.15)	0.98 (0.89–1.08)
40 +	2.02 (1.02–3.98)	0.98 (0.77–1.26)	1.09 (0.97–1.22)
Elixhauser score	1.03 (0.99–1.07)	1.02 (1.00–1.03)	1.00 (1.00–1.01)
Substances used			
Heroin	1.77 (0.54–5.76)	1.41 (0.96–2.08)	0.92 (0.78–1.08)
Rx opioid	0.71 (0.29–1.74)	0.82 (0.61–1.11)	1.16 (1.01–1.33)
Methadone	0.97 (0.13–7.34)	0.55 (0.24–1.26)	0.78 (0.58–1.06)
Alcohol	1.62 (0.89–2.95)	0.94 (0.73–1.22)	1.02 (0.91–1.14)
Stimulant	0.43 (0.22–0.85)	0.90 (0.73–1.11)	1.12 (1.02–1.24)
Injection opioid use	1.54 (0.77–3.08)	1.44 (1.14–1.81)	1.25 (1.13–1.39)
Opioid use frequency			
Not in last month	Ref	Ref	Ref
Use in past week-month	3.63 (1.33–9.95)	1.38 (1.00–1.89)	1.06 (0.92–1.21)
Daily	2.22 (0.84–5.89)	1.39 (1.05–1.83)	1.04 (0.93–1.17)
Prior inpatient medically supervised withdrawal	1.44 (0.75–2.77)	1.23 (0.94–1.59)	1.11 (0.98–1.26)
MOUD use	0.45 (0.23–0.89)	0.96 (0.78–1.20)	0.84 (0.76–0.93)
Prior all-cause ED/hospitalization	0.76 (0.41–1.40)	1.33 (1.08–1.64)	1.83 (1.67–2.01)
Prior opioid-related ED/hospitalization	2.16 (1.03–4.52)	1.72 (1.31–2.26)	0.94 (0.81–1.09)

Covariates for adjustment include: age, sex, race, substances used, injection drug use, frequency of opioid use, MOUD use, Elixhauser score, prior inpatient medically supervised withdrawal

*CI* confidence interval, *ED* emergency department encounter, *HR* hazard ratio, *MOUD* medications for opioid use disorder

**Table 3:** Opioid overdose, Opioid-related ED/Hospitalization, and treatment retention stratified by MOUD use (N = 3293)

	Opioid OD	Opioid-related ED/ Hospitalization	6-month retention	1-year retention
	Adjusted HR (95% CI)		Adjusted OR (95% CI)	
Overall				
Outpatient	Ref	Ref	Ref	Ref
Residential	1.39 (0.73–2.64)	1.02 (0.80–1.29)	1.71 (1.32–2.21)	1.12 (0.83–1.52)
Interaction p-value	0.851	0.005	0.001	0.006
MOUD				
Outpatient	Ref	Ref	Ref	Ref
Residential	1.21 (0.26–5.68)	1.59 (1.09–2.32)	0.75 (0.46–1.29)	0.65 (0.40–1.07)
No MOUD				
Outpatient	Ref	Ref	Ref	Ref
Residential	1.42 (0.71–2.82)	0.85 (0.64–1.11)	2.05 (1.56–2.71)	1.48 (1.03–2.12)

*CI* confidence interval, *ED* emergency department encounter, *HR* hazard ratio, *MOUD* medications for opioid use disorder, *OR* odds ratio

There was no association between index treatment setting and opioid-related ED/hospitalization (aHR 1.02; 95% CI 0.08–1.29) overall. However, the interaction with MOUD was significant (p = 0.005) suggesting that among individuals receiving MOUD, patients receiving residential care had a higher risk (aHR 1.59; 95% CI 1.09–2.32) of an opioid-related ED/hospitalization relative to outpatient care.

In both follow-up intervals, the risk for an all-cause ED/hospital admission was elevated for those receiving residential treatment [< 14 days: aHR 2.48 (95% CI 1.98–3.11); 14 + days: aHR 1.25 [95% CI 1.12–1.41)].

### Treatment retention

There were 1913 individuals (58% of original sample) with at least one year of Medicaid enrollment. Demographic variables and comorbidities were similar to the overall sample (Additional file 1: Table S6). Retention outcomes for these individuals are summarized in Table 4. Compared to those receiving outpatient treatment, individuals receiving residential treatment were more likely to be retained at 6-months (adjusted odds ratio [aOR] 1.71; 95% CI 1.32–2.21) with no difference at 12-months (aOR 1.12; 95% CI 0.83–1.52). For both retention outcomes, the MOUD interactions were significant, suggesting residential treatment was favored over outpatient treatment only for those without MOUD use.

**Table 4:** Multivariable logistic regression models of treatment retention among patients with one full year of enrollment (N = 1913)

	6-month retention	1-year retention
	Number retained (%)	
Treatment setting		
Outpatient (n = 1361)	618 (45.4%)	422 (31.0%)
Residential (n = 552)	234 (42.4%)	111 (21.1%)
	Adjusted OR (95% CI)	
Treatment setting		
Outpatient	Ref	Ref
Residential	1.71 (1.32–2.21)	1.12 (0.83–1.52)
Gender		
Male	Ref	Ref
Female	1.20 (0.97–1.47)	1.21 (0.95–1.53)
Race		
White	Ref	Ref
Black or African American	0.52 (0.23–1.17)	0.56 (0.23–1.37)
Alaska Native/American Indian	1.45 (0.81–2.60)	1.24 (0.64–2.39)
Other single race	0.79 (0.46–1.36)	0.77 (0.41–1.45)
Two or more races	1.16 (0.89–1.52)	1.12 (0.84–1.50)
Age		
18–29	Ref	Ref
30–39	1.11 (0.88–1.41)	0.99 (− 1.30)
40 +	1.08 (0.83–1.41)	0.96 (0.71–1.29)
Elixhauser score	0.99 (0.97–1.01)	0.99 (0.97–1.01)
Substances used		
Heroin	1.07 (0.73–1.57)	0.85 (0.56–1.29)
Rx opioid	0.99 (0.71–1.37)	0.89 (0.63–1.27)
Methadone	1.28 (0.64–2.56)	1.09 (0.54–2.17)
Alcohol	0.95 (0.73–1.22)	0.70 (0.51–0.97)
Stimulant	0.96 (0.77–1.20)	0.92 (0.71–1.20)
Injection opioid use	0.89 (0.70–1.14)	0.96 (0.73–1.27)
Opioid use frequency		
Not in last month	Ref	Ref
Use in past week-month	1.32 (0.96–1.80)	1.23 (0.84–1.80)
Daily	1.22 (0.93–1.59)	1.27 (0.92–1.74)
Prior inpatient medically supervised withdrawal	0.65 (0.48–0.88)	0.69 (0.47–1.00)
MOUD use	6.52 (5.11–8.33)	7.70 (5.93–9.98)
Prior all-cause ED/hospitalization	0.97 (0.78–1.21)	0.88 (0.69–1.12)
Prior opioid-related ED/hospitalization	1.39 (0.96–2.00)	1.43 (0.96–2.15)

Covariates for adjustment include: age, sex, race, substances used, injection drug use, frequency of opioid use, MOUD use, Elixhauser score, prior inpatient medically supervised withdrawal

*CI* confidence interval, *ED* emergency department encounter, *MOUD* medications for opioid use disorder, *OR* odds ratio

## Discussion

Residential treatment is often considered the highest intensity of treatment for individuals with OUD [40], and may by particularly important for those with unstable housing, co-morbid mental health conditions, or high medical need [41]. However, evidence supporting this assumption is mixed and has primarily focused on treatment completion, retention, and abstinence outcomes [9, 13]. Few studies have directly compared residential treatment with outpatient treatment for clinical outcomes such as overdose [20–22]. In this analysis, we used a linked Medicaid dataset to compare outcomes for individuals with OUD who received residential or outpatient treatment. After adjustment for a variety of physical, mental, and addiction-related comorbidities, we found that rates of overdose, opioid-related, and all-cause ED or hospitalizations were not reduced for individuals receiving residential treatment compared to those treated as an outpatient. While residential treatment was associated with higher retention at 6-months, this difference was not significant at 12-months. In stratified analyses, the benefits of residential treatment on retention appeared to be confined to those not receiving MOUD.

Historically, public perception has assumed residential treatment to be the gold standard, a view often endorsed by the addiction treatment community despite its greater cost and limited evidence [8, 13]. Efforts to further refine selection of patients most likely to benefit from residential treatment are likely to be eclipsed by increasing the use of MOUD in, and following, residential treatment. Opioid agonist treatment for OUD improves a variety of addiction-related outcomes and markedly reduces the risk of overdose and all-cause mortality [7, 42]. In our study, MOUD was associated with a 55% reduction in the risk of opioid overdose independent of treatment setting. About one-third of individuals receiving treatment were prescribed MOUD which is comparable to other reports and suggests missed opportunities for improving OUD treatment outcomes [1, 24].

This study adds to a mixed literature demonstrating the potential benefits of residential treatment for individuals with OUD with respect to treatment retention [13, 21, 22, 43]. Studies using SAMHSA TEDS data exclusively have generally shown that individuals entering residential facilities have higher treatment completion rates [43, 44]. Consistent with this literature, we found that residential treatment was associated with enhanced retention. While treatment completion is associated with improved some clinical and social outcomes, it is a surrogate indicator of improved addiction-related health outcomes. Moreover, OUD is now universally recognized as a chronic condition requiring long-term outpatient management. Although residential care was associated with improved retention in our study, it was not associated with improvements in overdose or other opioid-related outcomes. This largely comports with recent claims-based analyses that suggest outpatient treatment may be clinically superior to inpatient or residential treatment, especially when coupled with MOUD [21, 22].

Our subgroup analyses found that among individuals receiving MOUD, outpatient treatment was associated with improved opioid-related ED or hospitalizations compared to residential treatment. Using a similar retrospective cohort design, Morgan et al. found outpatient-based MOUD to be associated with improved rates of opioid overdose and all-cause admissions compared to inpatient treatment initiation [21]. Additional research is required to identify whether other subgroups of patients might benefit from residential treatment in the fentanyl era, such as those with a history of previous unsuccessful attempts at outpatient treatment, housing instability, and adolescents.

This study has several limitations. This was an observational study design, and findings are potentially affected by selection bias and confounding. Although we statistically adjusted for a wide variety of demographic, medical, and addiction-related covariates, our findings may be affected by residual confounding from unmeasured clinical and addiction-related characteristics. Further, the source data for this study was Medicaid administrative claims data, TEDS, and vital statistics data and missing data could affect our findings in uncertain ways. Because most outcomes were constructed using Medicaid data, gaps in Medicaid enrollment may also have introduced biases in how outcomes were measured. Although we measured opioid-related outcomes using ED and hospitalization administrative claims, non-fatal overdoses not treated in these settings could be missed. We were unable to link every treatment episode identified in the Medicaid dataset with a corresponding TEDS admission. This is likely due to under or inconsistent data submission by treatment providers and may further limit external validity [45]. Finally, study data was from one state Medicaid program and may not reflect treatment experiences or outcomes from other states or payers, such as those with commercial insurance. Despite this limitation, our findings are insightful because Oregon has among the highest burden of substance use in the nation, yet ranks last in treatment access [46].

## Conclusions

The findings of this study suggest residential treatment was not associated with improved outcomes relative to outpatient treatment. Although overall retention was enhanced for residential treatment in the short-term, this benefit was short-lived and limited to individuals not receiving MOUD. Importantly, MOUD use, independent of setting, was associated with a significant reduction in the risk for opioid overdose, underscoring the importance of MOUD across all treatment settings. In the absence of compelling evidence of benefit associated with residential treatment, expansion of treatment access should focus on broadening outpatient MOUD treatment capacity.

## Supplementary Information

**Additional file 1: Table S1**. CPT/HCPCS codes used to define initial treatment type. **Table S2**. Opioid use disorder diagnosis codes. **Table S3**. Elixhauser Comorbidity Conditions. **Table S4**. Medication for opioid use disorder codes.** Table S5**. Opioid-related ICD codes.** Table S6**. Patient and treatment characteristics of patients with one year of continuous Medicaid enrollment (n=1,913).

## Data Availability

The datasets generated and/or analyzed during the current study are not publicly available because of terms of data use agreements.

## Abbreviations

aHR: Adjusted hazard ratios
ASAM: American Society of Addiction Medicine
CPT: Current procedural terminology
ED: Emergency department
ICD: International Classification of Disease
MOUD: Medications for opioid use disorder
OUD: Opioid use disorder
SAMHSA: Substance Abuse and Mental Health Services Administration
TEDS: Treatment Episodes Dataset
XR-NTX: Extended-release naltrexone
