# Supplementary material for: Association between treatment setting and outcomes among oregon medicaid patients with opioid use disorder: a retrospective cohort study
Source: Addict Sci Clin Pract. 2022 Aug 19;17:45. doi: 10.1186/s13722-022-00318-1 (PMC9389731; doi:10.1186/s13722-022-00318-1)
Supplement: Supplementary file 1 — Additional file 1: Table S1. CPT/HCPCS codes used to define initial treatment type. Table S2. Opioid use disorder diagnosis codes. Table S3. Elixhauser Comorbidity Conditions. Table S4. Medication for opioid use disorder codes. Table S5. Opioid-related ICD codes. Table S6. Patient and treatment characteristics of patients with one year of continuous Medicaid enrollment (n=1,913). [file 13722_2022_318_MOESM1_ESM.docx]

Table S1: CPT/HCPCS codes used to define initial treatment type

| **Group** | **CPT / HCPCS code** |
| --- | --- |
| Residential | H0012 Alcohol And/Or Drug Services; Sub-Acute Detoxification (Residential Addiction Program Outpatient)  H0013 Alcohol And/Or Drug Services; Acute Detoxification (Residential Addiction Program Outpatient)  H0017 Behavioral Health; Residential (Hospital Residential Treatment Program), Without Room And Board, Per  H0018 Behavioral Health; Short-Term Residential (Non-Hospital Residential Treatment Program), Without Room  H0019 Behavioral Health; Long-Term Residential (Non-Medical, Non-Acute Care In A Residential Treatment Pro |
| Outpatient | H0001 Alcohol And/Or Drug Assessment  H0002 Behavioral Health Screening To Determine Eligibility For Admission To Treatment Program  H0004 Behavioral Health Counseling And Therapy, Per 15 Minutes  H0005 Alcohol And/Or Drug Services; Group Counseling By A Clinician  H0006 Alcohol and/or drug services; Case Management  H0007 Alcohol And/Or Drug Services; Crisis Intervention (Outpatient)  H0014 Alcohol And/Or Drug Services; Ambulatory Detoxification  H0015 Alcohol And/Or Drug Services; Intensive Outpatient (Treatment Program That Operates At Least 3 Hours  H0016 Alcohol And/Or Drug Services; Medical/Somatic (Medical Intervention In Ambulatory Setting)  H0022 Alcohol And/Or Drug Intervention Service (Planned Facilitation)  H0031 Mental Health Assessment, By Non-Physician  H0034 Medication Training And Support, Per 15 Minutes  H0035 Mental Health Partial Hospitalization, Treatment, Less Than 24 Hours  H0036 Community Psychiatric Supportive Treatment, Face-To-Face, Per 15 Minutes  H0037 Community Psychiatric Supportive Treatment Program, Per Diem  H0038 Self-help/peer services, per 15 min  H0039 Assertive Community Treatment, Face-To-Face, Per 15 Minutes  H0040 Assertive Community Treatment Program, Per Diem  H0047 Alcohol And/Or Other Drug Abuse Services, Not Otherwise Specified  H2000 Comprehensive Multidisciplinary Evaluation  H2001 Rehabilitation Program, Per 1/2 Day  H2010 Comprehensive Medication Services, Per 15 Minutes  H2011 Crisis Intervention Service, Per 15 Minutes  H2012 Behavioral Health Day Treatment, Per Hour  H2013 Psychiatric Health Facility Service, Per Diem  H2014 Skills Training And Development, Per 15 Minutes  H2015 Comprehensive Community Support Services, Per 15 Minutes  H2016 Comprehensive Community Support Services, Per Diem  H2017 Psychosocial Rehabilitation Services, Per 15 Minutes  H2018 Psychosocial Rehabilitation Services, Per Diem  H2019 Therapeutic Behavioral Services, Per 15 Minutes  H2020 Therapeutic Behavioral Services, Per Diem  H2023 Supported Employment/education, per 15 min  H2035 Alcohol And/Or Other Drug Treatment Program, Per Hour  H2036 Alcohol And/Or Other Drug Treatment Program, Per Diem |
|  | 90791 Psychiatric diagnostic evaluation  90792 Psychiatric diagnostic evaluation with medical services  90832 Psychotherapy, 30 minutes with patient and/or family member  90833 Psychotherapy, 30 minutes with patient and/or family member when performed with an E/M service  90834 Psychotherapy, 45 minutes with patient and/or family member  90836 Psychotherapy, 45 minutes with patient and/or family member when performed with an E/M service  90837 Psychotherapy, 60 minutes with patient and/or family member.  90838 Psychotherapy, 60 minutes with patient and/or family member when performed with an E/M service  90839 Psychotherapy for crisis, first 60 minutes  90840 Psychotherapy for crisis (each additional 30 minutes) - list separately in addition to primary service CPT code.  90853 Group psychotherapy  90863 Under Other Psychiatric Services or Procedures  90875 Under Other Psychiatric Services or Procedures  90876 Under Other Psychiatric Services or Procedures  G0396 Alcohol and/or substance (other than tobacco) abuse structured assessment (e.g., audit, dast), and brief intervention 15 to 30 minutes  G0397 Alcohol and/or substance (other than tobacco) abuse structured assessment (e.g., audit, dast), and intervention, greater than 30 minutes  M0064 Brief office visit for the sole purpose of monitoring or changing drug prescriptions used in the treatment of mental psychoneurotic and personality disorders  S0201 Partial hospitalization services, less than 24 hours, per diem  S9480 Intensive outpatient psychiatric services, per diem  S9484 Crisis intervention mental health services, per diem  S9485 Crisis intervention mental health services, per diem  T1006 Alcohol and/or substance abuse services, family/couple counseling  T1012 Alcohol and/or substance abuse services, skills development |
| Inpatient medically supervised withdrawal (i.e. detoxification) | H0008 Alcohol And/Or Drug Services; Sub-Acute Detoxification (Hospital Inpatient)  H0009 Alcohol And/Or Drug Services; Acute Detoxification (Hospital Inpatient)  H0010 Alcohol And/Or Drug Services; Sub-Acute Detoxification (Residential Addiction Program Inpatient)  H0011 Alcohol And/Or Drug Services; Acute Detoxification (Residential Addiction Program Inpatient) |

Table S2: Opioid use disorder diagnosis codes

| **ICD** | **Diagnosis** | **Codes** |
| --- | --- | --- |
| ICD9 | Opioid dependence  Opioid or other drug dependence  Nondependent drug abuse | 304.00–304.03  304.70–304.73  305.50–305.53 |
| ICD10 | Opioid abuse, dependence  Opioid use | F11.1x – F11.2x  F11.9x |

Table S3: Elixhauser Comorbidity Conditions

| **Condition** |
| --- |
| Acquired immune deficiency syndrome |
| Alcohol abuse |
| Deficiency anemias |
| Autoimmune conditions |
| Chronic blood loss anemia |
| Leukemia |
| Lymphoma |
| Metastatic cancer |
| Solid tumor without metastasis, in situ |
| Solid tumor without metastasis, malignant |
| Cerebrovascular disease |
| Coagulopathy |
| Dementia |
| Depression |
| Diabetes with chronic complications |
| Diabetes without chronic complications |
| Drug abuse |
| Heart failure |
| Hypertension, complicated |
| Hypertension, uncomplicated |
| Liver disease, mild |
| Liver disease, moderate to severe |
| Chronic pulmonary disease |
| Neurological disorders affecting movement |
| Other neurological disorders |
| Seizures and epilepsy |
| Obesity |
| Paralysis |
| Peripheral vascular disease |
| Psychoses |
| Pulmonary circulation disease |
| Renal failure, moderate |
| Renal failure, severe |
| Hypothyroidism |
| Other thyroid disorders |
| Peptic ulcer with bleeding |
| Valvular disease |
| Weight loss |

Table S4: Medication for opioid use disorder codes

| **Clinic Administered medication** | |
| --- | --- |
| Methadone | H0020 Alcohol And/Or Drug Services; Methadone Administration,  H0033 Oral Medication Administration, direct observation |
| Buprenorphine | J0571 BUPRENORPHINE ORAL 1 MG  J0572 BUPRENORPHINE/NALOXONE ORAL </=TO 3 MG BPN  J0573 BUPRENORPHNE/NALOXONE ORAL >3 MG BUT </=6 MG BPN  J0574 BUPRENORPHINE/NLX ORAL >6 MG BUT </=TO 10 MG BPN  J0575 BUPRENORPHINE/NALOXONE ORAL >10 MG BUPRENORPHINE  J0570 Buprenorphine implant, 74.2 mg (Probuphine)  Q9991 Injection, buprenorphine extended-release (Sublocade), less than or equal to 100 mg  Q9992 Injection, buprenorphine extended-release (Sublocade), greater than 100 mg |
| XR-NTX | J2315 |
| **Outpatient pharmacy medications** | |
|  | \| **NDC** \| **Brand name** \| **Strength** \| **formulation** \| \| --- \| --- \| --- \| --- \| \| 00054017613 \| BUPRENORPHINE HCL \| 2 mg \| TAB SUBL \| \| 00054017713 \| BUPRENORPHINE HCL \| 8 mg \| TAB SUBL \| \| 00054018813 \| BUPRENORPHINE-NALOXONE \| 2 mg-0.5 mg \| TAB SUBL \| \| 00054018913 \| BUPRENORPHINE-NALOXONE \| 8 mg-2 mg \| TAB SUBL \| \| 00093537856 \| BUPRENORPHINE HCL \| 2 mg \| TAB SUBL \| \| 00093537956 \| BUPRENORPHINE HCL \| 8 mg \| TAB SUBL \| \| 00093572056 \| BUPRENORPHINE-NALOXONE \| 2 mg-0.5 mg \| TAB SUBL \| \| 00093572156 \| BUPRENORPHINE-NALOXONE \| 8 mg-2 mg \| TAB SUBL \| \| 00228315303 \| BUPRENORPHINE HCL \| 8 mg \| TAB SUBL \| \| 00228315403 \| BUPRENORPHINE-NALOXONE \| 2 mg-0.5 mg \| TAB SUBL \| \| 00228315503 \| BUPRENORPHINE-NALOXONE \| 8 mg-2 mg \| TAB SUBL \| \| 00228315603 \| BUPRENORPHINE HCL \| 2 mg \| TAB SUBL \| \| 00378092393 \| BUPRENORPHINE HCL \| 2 mg \| TAB SUBL \| \| 00378092493 \| BUPRENORPHINE HCL \| 8 mg \| TAB SUBL \| \| 00406192303 \| BUPRENORPHINE-NALOXONE \| 2 mg-0.5 mg \| TAB SUBL \| \| 00406192403 \| BUPRENORPHINE-NALOXONE \| 8 mg-2 mg \| TAB SUBL \| \| 12496120201 \| SUBOXONE \| 2 mg-0.5 mg \| FILM \| \| 12496120203 \| SUBOXONE \| 2 mg-0.5 mg \| FILM \| \| 12496120401 \| SUBOXONE \| 4 mg-1 mg \| FILM \| \| 12496120403 \| SUBOXONE \| 4 mg-1 mg \| FILM \| \| 12496120801 \| SUBOXONE \| 8 mg-2 mg \| FILM \| \| 12496120803 \| SUBOXONE \| 8 mg-2 mg \| FILM \| \| 12496121201 \| SUBOXONE \| 12 mg-3 mg \| FILM \| \| 12496121203 \| SUBOXONE \| 12 mg-3 mg \| FILM \| \| 12496127802 \| SUBUTEX \| 2 mg \| TAB SUBL \| \| 12496128302 \| SUBOXONE \| 2 mg-0.5 mg \| TAB SUBL \| \| 12496130602 \| SUBOXONE \| 8 mg-2 mg \| TAB SUBL \| \| 12496131002 \| SUBUTEX \| 8 mg \| TAB SUBL \| \| 16590066630 \| SUBOXONE \| 2 mg-0.5 mg \| TAB SUBL \| \| 35356000407 \| SUBOXONE \| 8 mg-2 mg \| TAB SUBL \| \| 35356000430 \| SUBOXONE \| 8 mg-2 mg \| TAB SUBL \| \| 35356055530 \| BUPRENORPHINE HCL \| 2 mg \| TAB SUBL \| \| 35356055630 \| BUPRENORPHINE HCL \| 8 mg \| TAB SUBL \| \| 42291017430 \| BUPRENORPHINE-NALOXONE \| 2 mg-0.5 mg \| TAB SUBL \| \| 42291017530 \| BUPRENORPHINE-NALOXONE \| 8 mg-2 mg \| TAB SUBL \| \| 43063018407 \| SUBOXONE \| 8 mg-2 mg \| TAB SUBL \| \| 43063018430 \| SUBOXONE \| 8 mg-2 mg \| TAB SUBL \| \| 49999039507 \| SUBOXONE \| 2 mg-0.5 mg \| TAB SUBL \| \| 49999039515 \| SUBOXONE \| 2 mg-0.5 mg \| TAB SUBL \| \| 49999039530 \| SUBOXONE \| 2 mg-0.5 mg \| TAB SUBL \| \| 49999063830 \| SUBUTEX \| 2 mg \| TAB SUBL \| \| 49999063930 \| SUBUTEX \| 8 mg \| TAB SUBL \| \| 50383092493 \| BUPRENORPHINE HCL \| 2 mg \| TAB SUBL \| \| 50383093093 \| BUPRENORPHINE HCL \| 8 mg \| TAB SUBL \| \| 52959030430 \| SUBOXONE \| 8 mg-2 mg \| TAB SUBL \| \| 52959074930 \| SUBOXONE \| 2 mg-0.5 mg \| TAB SUBL \| \| 54123091430 \| ZUBSOLV \| 1.4 mg-0.36 mg \| TAB SUBL \| \| 54123095730 \| ZUBSOLV \| 5.7 mg-1.4 mg \| TAB SUBL \| \| 54123098630 \| ZUBSOLV \| 8.6 mg-2.1 mg \| TAB SUBL \| \| 54569549600 \| SUBOXONE \| 2 mg-0.5 mg \| TAB SUBL \| \| 54569573900 \| SUBOXONE \| 8 mg-2 mg \| TAB SUBL \| \| 54569573901 \| SUBOXONE \| 8 mg-2 mg \| TAB SUBL \| \| 54569573902 \| SUBOXONE \| 8 mg-2 mg \| TAB SUBL \| \| 54569639900 \| SUBOXONE \| 8 mg-2 mg \| FILM \| \| 54569640800 \| BUPRENORPHINE-NALOXONE \| 8 mg-2 mg \| TAB SUBL \| \| 54868570700 \| SUBOXONE \| 8 mg-2 mg \| TAB SUBL \| \| 54868570701 \| SUBOXONE \| 8 mg-2 mg \| TAB SUBL \| \| 54868570702 \| SUBOXONE \| 8 mg-2 mg \| TAB SUBL \| \| 54868570703 \| SUBOXONE \| 8 mg-2 mg \| TAB SUBL \| \| 54868570704 \| SUBOXONE \| 8 mg-2 mg \| TAB SUBL \| \| 54868575000 \| SUBOXONE \| 2 mg-0.5 mg \| TAB SUBL \| \| 55045378403 \| SUBOXONE \| 8 mg-2 mg \| TAB SUBL \| \| 55700014730 \| SUBOXONE \| 8 mg-2 mg \| FILM \| \| 55700018430 \| BUPRENORPHINE-NALOXONE \| 2 mg-0.5 mg \| TAB SUBL \| \| 59385001201 \| BUNAVAIL \| 2.1 mg-0.3 mg \| FILM \| \| 59385001230 \| BUNAVAIL \| 2.1 mg-0.3 mg \| FILM \| \| 59385001401 \| BUNAVAIL \| 4.2 mg-0.7 mg \| FILM \| \| 59385001430 \| BUNAVAIL \| 4.2 mg-0.7 mg \| FILM \| \| 59385001601 \| BUNAVAIL \| 6.3 mg-1 mg \| FILM \| \| 59385001630 \| BUNAVAIL \| 6.3 mg-1 mg \| FILM \| \| 63629402801 \| SUBOXONE \| 2 mg-0.5 mg \| TAB SUBL \| \| 63629403401 \| SUBOXONE \| 8 mg-2 mg \| TAB SUBL \| \| 63629403402 \| SUBOXONE \| 8 mg-2 mg \| TAB SUBL \| \| 63629403403 \| SUBOXONE \| 8 mg-2 mg \| TAB SUBL \| \| 63874108403 \| SUBOXONE \| 8 mg-2 mg \| TAB SUBL \| \| 63874108503 \| SUBOXONE \| 2 mg-0.5 mg \| TAB SUBL \| \| 63874117303 \| SUBUTEX \| 8 mg \| TAB SUBL \| \| 65162041503 \| BUPRENORPHINE-NALOXONE \| 8 mg-2 mg \| TAB SUBL \| \| 65162041603 \| BUPRENORPHINE-NALOXONE \| 2 mg-0.5 mg \| TAB SUBL \| \| 66336001630 \| SUBOXONE \| 8 mg-2 mg \| TAB SUBL \| \| 68071138003 \| SUBOXONE \| 8 mg-2 mg \| TAB SUBL \| \| 68071151003 \| SUBOXONE \| 2 mg-0.5 mg \| TAB SUBL \| \| 68258299903 \| SUBOXONE \| 2 mg-0.5 mg \| TAB SUBL \| \| 68308020230 \| BUPRENORPHINE HCL \| 2 mg \| TAB SUBL \| \| 68308020830 \| BUPRENORPHINE HCL \| 8 mg \| TAB SUBL \| |

Table S5: Opioid-related ICD codes

| **Opioid overdose** | | |
| --- | --- | --- |
| ICD9 | *Poisoning by:*  opium  heroin  methadone  other opiates and related narcotics  *Accidental poisoning by:*  heroin  methadone  other opiates and related narcotics | 96500  96501  96502  96509  E8500  E8501  E8502 |
| ICD10 | *Poisoning by:*  Opium  heroin  Natural or semi-synthetic opioids  Methadone  Synthetic opioids, other than methadone  Other and unspecified narcotics | T400  T401  T402  T403  T404  T406 |
| **Non-overdose opioid-related** | | |
| ICD9 | Opioid dependence  Opioid or other drug dependence  Nondependent drug abuse | 304.00–304.03  304.70–304.73  305.50–305.53 |
| ICD10 | Opioid abuse, dependence  Opioid use | F11.1x – F11.2x  F11.9x |

Table S6: Patient and treatment characteristics of patients with one year of continuous Medicaid enrollment (n=1,913)

|  | Outpatient (n=1361) | Residential (n=552) | Total (n=1913) |
| --- | --- | --- | --- |
| Female | 609 (44.7%) | 277 (50.2%) | 886 (46.3%) |
| Race |  |  |  |
| White | 1006 (73.9%) | 402 (72.8%) | 1408 (73.6%) |
| Black or African American | 26 (1.9%) | 6 (1.1%) | 32 (1.7%) |
| Alaska Native/American Indian | 39 (2.9%) | 19 (3.4%) | 58 (3.0%) |
| Other single race | 54 (4.0%) | 18 (3.3%) | 72 (3.8%) |
| Two or more races | 236 (17.3%) | 107 (19.4%) | 343 (17.9%) |
| Ethnicity |  |  |  |
| Hispanic or Latino | 89 (6.5%) | 56 (10.1%) | 145 (7.6%) |
| Not Hispanic or Latino | 1209 (88.8%) | 486 (88.0%) | 1695 (88.6%) |
| Unknown/Not Reported | 63 (4.6%) | 10 (1.8%) | 73 (3.8%) |
| Age |  |  |  |
| 18-29 | 537 (39.5%) | 294 (53.3%) | 831 (43.4%) |
| 30-39 | 434 (31.9%) | 171 (31.0%) | 605 (31.6%) |
| 40+ | 390 (28.7%) | 87 (15.8%) | 477 (24.9%) |
| Elixhauser conditions (Only those with prevalence > 3% presented) | | | |
| Alcohol misuse | 258 (19.0%) | 149 (27.0%) | 407 (21.3%) |
| Anemia deficiency | 48 (3.5%) | 17 (3.1%) | 65 (3.4%) |
| Chronic pulmonary disease | 140 (10.3%) | 56 (10.1%) | 196 (10.3%) |
| Depression | 205 (15.1%) | 140 (25.4%) | 345 (18.0%) |
| Drug abuse | 1360 (100.0%) | 552 (100.0%) | 1912 (100.0%) |
| Hypertension | 166 (12.2%) | 48 (8.7%) | 214 (11.2%) |
| Liver disease | 73 (5.4%) | 41 (7.4%) | 114 (6.0%) |
| Fluid and electrolyte disorders | 68 (5.0%) | 38 (6.9%) | 106 (5.5%) |
| Other neurological disorders | 95 (7.0%) | 57 (10.3%) | 152 (7.9%) |
| Psychoses | 113 (8.3%) | 103 (18.7%) | 216 (11.3%) |
| Elixhauser score, mean (SD) | -6.8 (5.0) | -7.4 (5.6) | -7.0 (5.2) |
| MOUD use* |  |  |  |
| Any | 569 (41.8%) | 82 (14.9%) | 651 (34.0%) |
| Methadone | 111 (8.2%) | 41 (7.4%) | 152 (7.9%) |
| Buprenorphine | 461 (33.9%) | 41 (7.4%) | 502 (26.2%) |
| XR naltrexone | 0 (0.0%) | 3 (0.5%) | 3 (0.2%) |
| Substance used |  |  |  |
| Heroin | 913 (67.1%) | 429 (77.7%) | 1342 (70.2%) |
| Prescription opioids | 578 (42.5%) | 170 (30.8%) | 748 (39.1%) |
| Non-prescription methadone | 38 (2.8%) | 8 (1.4%) | 46 (2.4%) |
| Alcohol | 308 (22.6%) | 103 (18.7%) | 411 (21.5%) |
| Stimulant | 403 (29.6%) | 273 (49.5%) | 676 (35.3%) |
| Injection opioid use | 612 (45.0%) | 323 (58.5%) | 935 (48.9%) |
| Frequency of use |  |  |  |
| None in past month | 311 (22.9%) | 111 (20.1%) | 422 (22.1%) |
| Use in past week/month | 182 (19.0%) | 134 (24.3%) | 392 (20.5%) |
| Daily | 792 (58.2%) | 307 (55.6%) | 1099 (57.4%) |
| Prior inpatient medically supervised withdrawal | 92 (6.8%) | 235 (42.6%) | 327 (17.1%) |
| Prior all-cause ED/hospitalization | 685 (50.3% | 338 (61.2%) | 1023 (53.3% |
| Prior opioid-related ED/hospitalization | 93 (6.8%) | 80 (15.4%) | 173 (9.0%) |
